# Retreatment with immunotherapy in a patient with hepatocellular carcinoma who received immune checkpoint inhibitors after primary curative treatment: a case report

**DOI:** 10.3389/fonc.2024.1321195

**Published:** 2024-04-05

**Authors:** Tao Wang, Fei Tang, Fenghui Li, Weili Yin, Jing Liang

**Affiliations:** Department of Gastroenterology and Hepatology, The Third Central Hospital of Tianjin, Tianjin Key Laboratory of Extracorporeal Life Support for Critical Diseases, Artificial Cell Engineering Technology Research Center, Tianjin Institute of Hepatobiliary Disease, Tianjin, China

**Keywords:** hepatocellular carcinoma, immune checkpoint inhibitors, curative treatment, early recurrence, retreatment

## Abstract

Hepatocellular carcinoma (HCC) presents a malignant pathology known for its high early recurrence rate following curative treatment, significantly impacting patient prognosis. Currently, effective strategies to mitigate early HCC recurrence remain undetermined. In this report, we document a case of HCC managed with curative radiofrequency ablation (RFA), particularly in a patient facing a high risk of early recurrence due to a substantial tumor size. In an effort to forestall recurrence, immune checkpoint inhibitors (ICIs) were preemptively administered for 6 months post-RFA. Despite this, early recurrence ensued upon ICIs cessation. Traditionally, the approach to advanced HCC has been conservative, yet recent years have seen promising outcomes with ICIs in advanced HCC. However, research on ICIs retreatment is limited. In the short term, this patient experienced widespread metastases post-ICIs discontinuation, yet exhibited prompt regression upon ICIs reinitiation. Notably, this represents the initial documented instance of employing ICIs to forestall recurrence subsequent to curative RFA in HCC. Following ICIs discontinuation, diffuse recurrence with multiple metastases emerged, with successful resolution upon ICIs retreatment.

## Background

Hepatocellular carcinoma (HCC) presently stands as the sixth most prevalent malignancy and the third leading cause of cancer-related mortality, presenting a profound threat to patients’ well-being and longevity (1). Recurring readily even following curative intervention, approximately 70% of patients encounter recurrence within 5 years (2). Early recurrence manifests within 2 years post-curative treatment, contributing to about 70% of HCC recurrences within this 5-year period, significantly impacting patients’ survival rates (3). At present, there exists no established efficacious approach to forestall early HCC recurrence subsequent to curative treatment.

Immune checkpoint inhibitors (ICIs) have profoundly transformed the landscape of cancer therapy, elevating survival prospects for individuals contending with advanced cancer. Several clinical trials and empirical evidence have underscored the extension of survival among patients with inoperable liver cancer due to ICIs (4, 5). More recently, the less ability of single-agent ICIs to improve overall survival (OS) in phase III clinical trials has led to the development of combination approaches, such as anti-programmed cell death-1 (PD-1)/anti-programmed cell death ligand 1 (PD-L1) plus anti-vascular endothelial growth factor (VEGF), or tyrosine kinase inhibitors (TKIs), or anti-cytotoxic T-lymphocyte associated antigen-4 (CTLA-4) (6). IMbrave 150 demonstrated that atezolizumab plus bevacizumab led to significantly improved OS compared with sorafenib in patients with unresectable HCC (7). With the increasing utilization of ICIs in advanced HCC, an escalating number of patients are encountering challenges related to drug discontinuation, recurrence, and subsequent treatment. Limited studies have addressed the retreatment with ICIs in patients with solid tumors (8).

In this instance, we present a case of HCC managed with radiofrequency ablation (RFA) despite a substantial tumor size and a heightened vulnerability to early recurrence. Following RFA, the patient underwent ICIs therapy as a preventive measure against recurrence. Regrettably, the patient encountered early recurrence subsequent to the discontinuation of ICIs, resulting in diffuse liver recurrence and multiple metastases in the lungs and adrenal glands. Notwithstanding the patient’s prior ICIs treatment history, the response to retreatment with atezolizumab combined with bevacizumab was notably favorable.

## Case presentation

**Figure 1:**
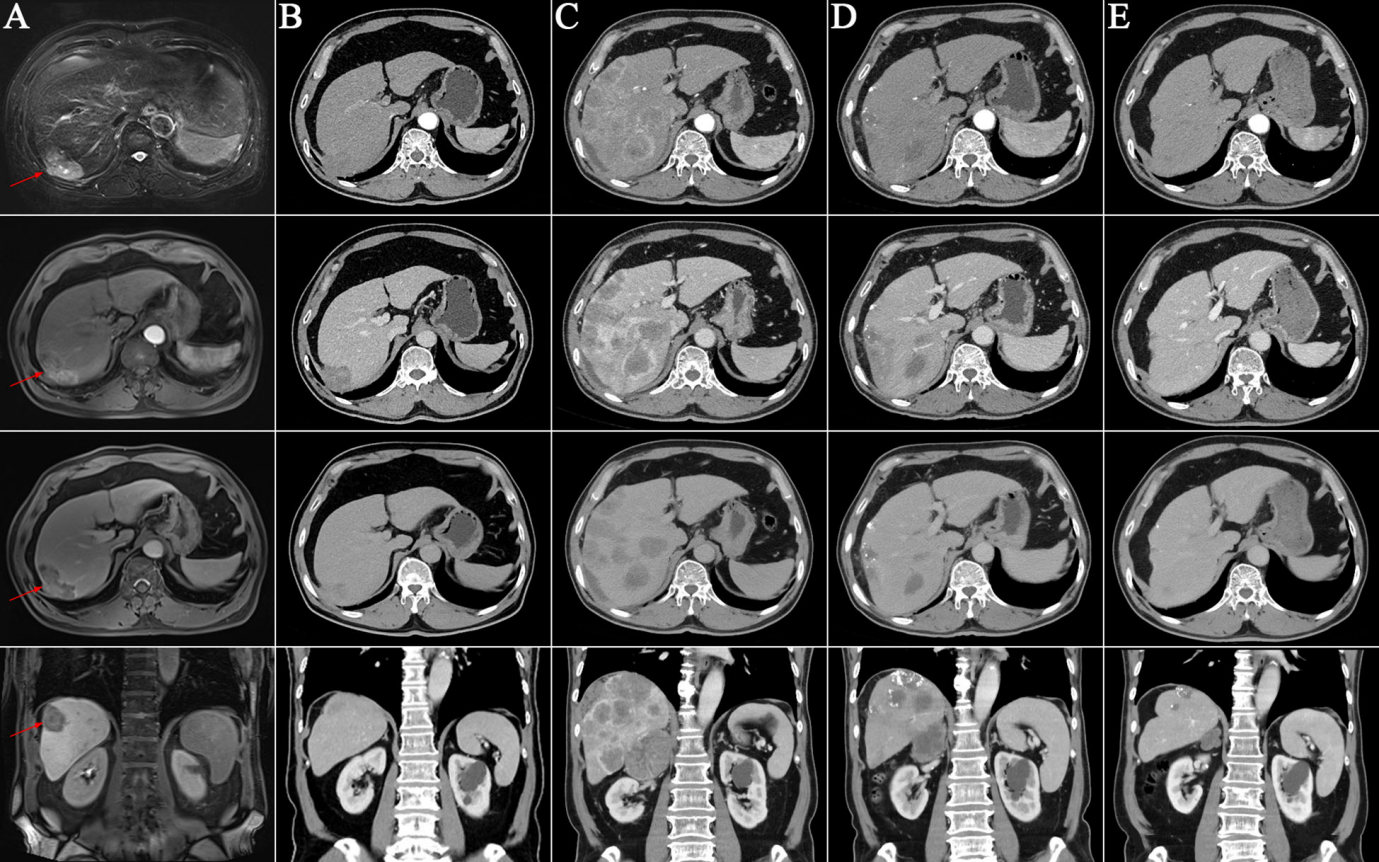
Enhanced liver imagings at different time points. **(A)** The T2-phase, arterial phase, portal vein phase, and coronal image of the enhanced liver magnetic resonance imaging on May 26, 2020. It revealed a mass in the right hepatic lobe (red arrows); **(B-E)** The arterial phase, portal vein phase, equilibrium phase and coronal image of the enhanced liver computed tomography images; **(B)** No signs of tumor on October 21, 2021; **(C)** Diffuse recurrence in the liver on May 7, 2022; **(D)** After two cycles of retreatment of immunotherapy on July 21, 2022. Significant reduction of liver lesions; **(E)** CT indicated no enhancing tumor lesions in the liver on September 15, 2023.

**Figure 2:**
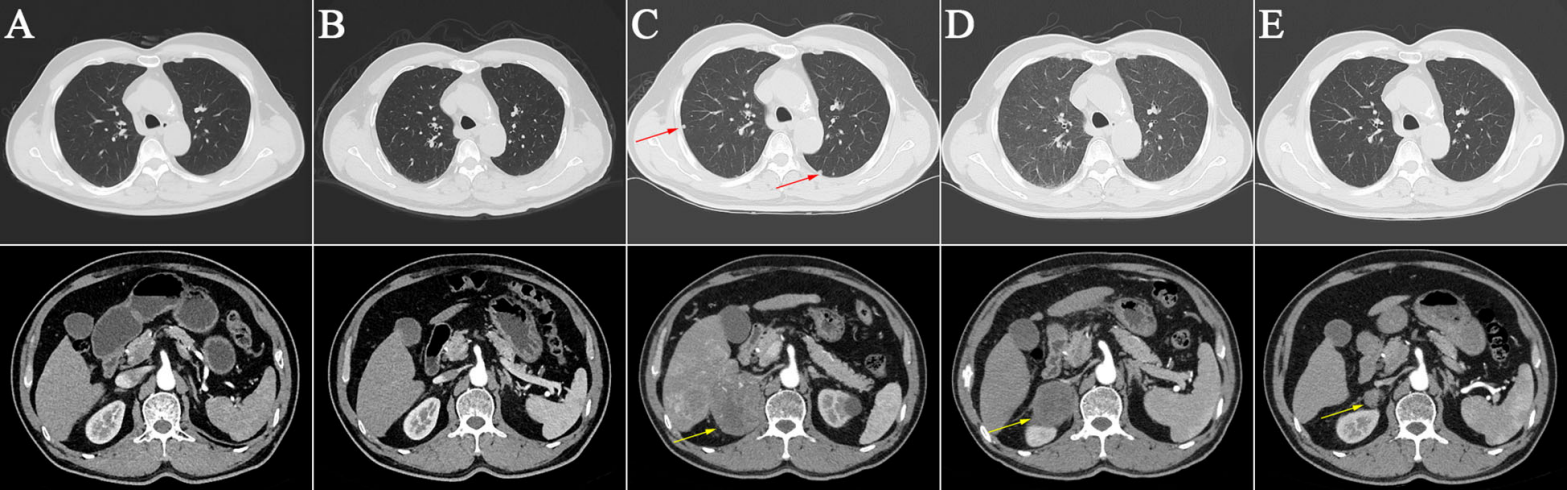
Abdominal and pulmonary computerized tomography imagings at different time points. **(A)** Base line on May 26, 2020; **(B)** No pulmonary and adrenal metastasis on October 21, 2021; **(C)** Pulmonary and adrenal metastasis on May 7, 2022 (red and yellow arrows); **(D)** Interstitial pneumoni on July 21, 2022; **(E)** No pulmonary lesions and significantly reduced adrenal lesions on September 15, 2023 (yellow arrows).

On May 26, 2020, a 70-year-old man presented at our facility, reporting a six-month history of discomfort in the right upper abdomen. With a four-decade record of alcohol misuse equivalent to 140 g/day of ethanol, the patient also disclosed a history of hypertension and smoking but no indications of diabetes, chronic viral hepatitis, autoimmune conditions, or blood transfusion. Enhanced magnetic resonance imaging (MRI) unveiled a 6.5×3.0 cm tumor in the right hepatic lobe (**Figure 1A**). Abdominal and pulmonary computerized tomography imagings showed no metastasis lesions (**Figure 2A**). Assessments for hepatitis viruses, autoimmune liver conditions, and metabolic liver disorders such as hepatolenticular degeneration and hemochromatosis yielded negative results. The alpha-fetoprotein (AFP) level stood at 170.30 ng/mL, and liver function tests proved nearly normal (**Supplementary Table S1**). Subsequent diagnosis included primary HCC (Barcelona Clinic Liver Cancer B, Eastern Cooperative Oncology Group Performance Score 0), alcoholic cirrhosis (Child-Pugh grade A), and hypertension. The patient underwent transarterial chemoembolization (TACE) and RFA a month later. Liver pathology findings suggested moderately differentiated HCC. After RFA, the patient’s abdominal discomfort disappeared.

Due to the patient’s heightened susceptibility to early HCC recurrence, immunotherapy with ICIs was initiated on August 25, 2020, administered once every 3 weeks for a total duration of 6 months. After two cycles of immunotherapy, the patient developed a nonspecific maculopapular rash and exhibited positive urine protein. Addressing these concerns, topical hormonal medications and oral antihistamines were prescribed, leading to the amelioration of the rash without exacerbating the urine protein status. The patient received Dulvalizumab, and it remains unclear whether bevacizumab was included as part of a clinical trial, the results of which are not publicly available. The last session of immunotherapy took place on January 19, 2021, with contrast-enhanced computed tomography (CT) scans conducted every 3 months throughout the treatment period, revealing no signs of HCC recurrence. Following cessation of ICIs, the rash subsided, and subsequent urine protein tests returned negative. By October 21, 2021, contrast-enhanced CT scans exhibited no evidence of HCC recurrence and no metastasis lesions (**Figures 1B**, **2B**).

**Figure 3:**
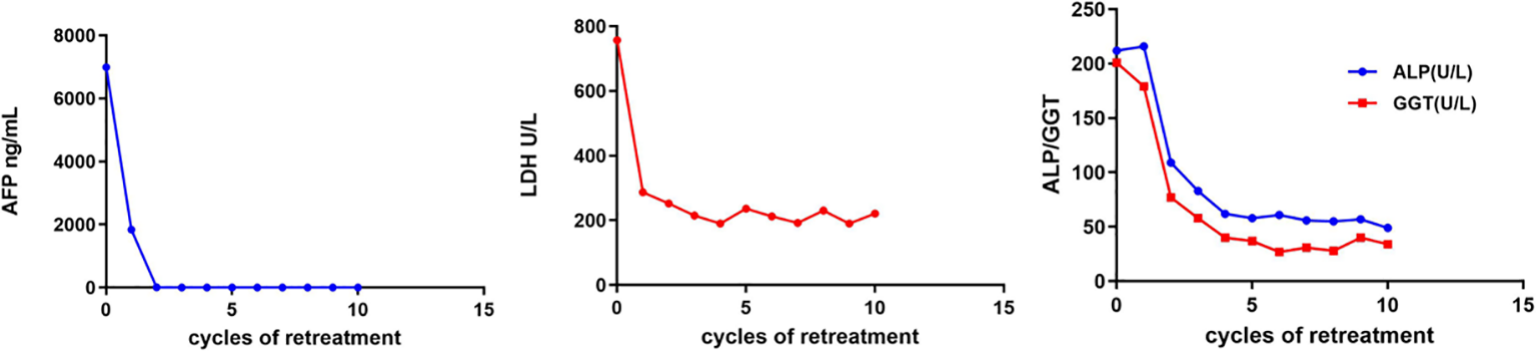
Changes of clinical indicators. AFP, alpha-fetoprotein; LDH, lactate dehydrogenase; ALP, alkaline phosphatase; GGT, gamma-glutamyltransferase.

**Figure 4:**
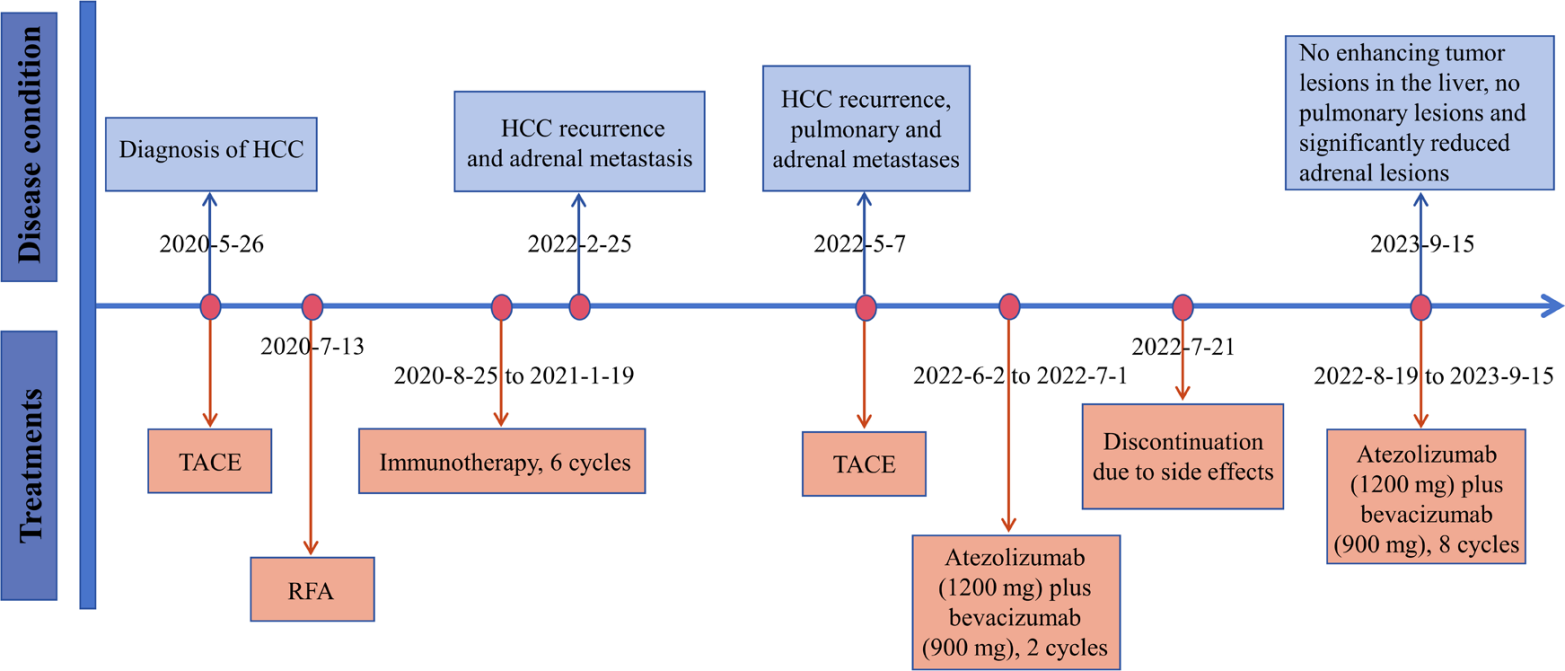
The timeline with therapy and disease status. HCC, hepatocellular carcinoma; TACE, transarterial chemoembolization; RFA, radiofrequency ablation.

On February 25, 2022, the patient got right upper abdominal discomfort again and intensive MRI revealed the reappearance of HCC along with adrenal metastasis; however, the patient declined further intervention. Subsequently, on May 7, 2022, he was hospitalized due to HCC recurrence accompanied by pulmonary and adrenal metastases (**Figures 1C**, **2C**). Evaluation of liver function exhibited mild elevations in alkaline phosphatase (ALP), gamma-glutamyltransferase (GGT), and lactate dehydrogenase (LDH) levels (**Supplementary Table S1**). With an AFP level surpassing the detection limit of 1,210 ng/mL, the patient underwent TACE. Moreover, on June 2, 2022, the patient commenced a renewed course of immunotherapy (atezolizumab 1200 mg combined with 900 mg of bevacizumab once every 3 weeks). Following two cycles of immunotherapy, he progressively developed post-exertional wheezing, and chest CT revealed the presence of interstitial pneumonia (**Figure 2D**), leading to a grade 2 immune pneumonia diagnosis. Consequently, immunotherapy was discontinued. Concurrently, contrast-enhanced CT illustrated a noteworthy reduction in the intrahepatic, pulmonary, and adrenal lesions (**Figures 1D**, **2D**). By August 19, 2022, the patient’s wheezing disappeared and a repeat chest CT at an alternative medical facility demonstrated improvements in inflammation, warranting the resumption of immunotherapy. Notably, the AFP, LDH, ALP, and GGT levels normalized (**Figure 3**). Subsequent to a further contrast-enhanced CT on September 15, 2023, no enhancing tumor lesions in the liver, no pulmonary lesions, and significantly decreased adrenal lesions were observed (**Figures 1E**, **2E**). Currently, the abdominal discomfort has significantly relieved. The patient’s course is shown in **Figure 4**.

## Discussion

The postoperative resurgence of HCC is believed to be associated with the intrinsic biological attributes of the HCC, including tumor size, capsule integrity, differentiation level, and the presence of vascular invasion (9, 10). Early recurring nodules demonstrate heightened malignancy, increased metastatic potential, greater likelihood of relapse, and consequently, a less favorable prognosis compared to instances of late recurrence (recurrence occurring >2 years following curative treatment of HCC) (11). Tumor size stands out as a pivotal factor influencing early recurrence, with patients harboring tumors >3.5 cm in diameter facing a heightened risk of postoperative resurgence (12). In the case of this patient, the tumor was large (diameter surpassed 5 cm) and relatively poorly differentiated, thus placing him at a heightened risk for early recurrence following RFA.

There is currently no established standard treatment for mitigating the risk of recurrence subsequent to curative treatment for HCC. The efficacy of adjuvant therapy in reducing HCC recurrence after curative treatment, especially early recurrence, remains a topic of debate. Such therapies encompass a range of interventions including chemotherapy, radiotherapy, TACE, molecular targeted therapy, immunotherapy, vitamin K2 analogs, retinoids, and antiviral therapy (13, 14).

At present, ICIs stand as the primary immunotherapeutic choice effective across a broad spectrum of cancers, particularly those resistant to chemotherapy (15, 16). These immune checkpoints entail co-inhibitory receptors on T cells alongside their ligands on tumor and stromal cells within the tumor microenvironment (17). ICIs function by averting T-cell inactivation through the blockade of interactions between checkpoint proteins and their ligands, thereby eliciting anti-tumor effects (18). Key immune checkpoints of interest include programmed cell death-1 (PD-1), programmed cell death ligand 1 (PD-L1), and cytotoxic T-lymphocyte associated antigen-4 (CTLA-4). In the context of unresectable HCC, single-agent anti-PD-1 demonstrates an efficacy of approximately 20%, while combination therapy exhibits even higher efficacy. ICIs, whether administered individually or in combination, now represent the cornerstone of systemic therapy for patients with advanced HCC (19, 20). Nevertheless, the safety of ICIs in early- and mid-stage HCC, along with their efficacy in preventing HCC recurrence, requires validation through extensive clinical trials and further investigations.

Greten et al. documented the activation of tumor-specific T and dendritic cells subsequent to RFA treatment in animal models (21). Consequently, local treatment holds the potential to ameliorate T-cell immunosuppression. In an HCC animal model, Hepa1-6 mice subjected to microwave ablation and anti-PD-1/anti-CTLA-4 demonstrated heightened intratumoral infiltration of Th1 cells, prolonged survival, and impeded tumor recurrence (22). These investigations offer a theoretical foundation for the amalgamation of local treatment with immunotherapy among HCC patients. The ongoing IMbrave 050 study explores the implementation of ICIs following curative treatment to forestall HCC recurrence (23).

As our patient faced a heightened risk of early HCC recurrence following RFA, he was enrolled in a clinical investigation involving ICIs aimed at averting tumor reappearance. Throughout the trial period, the patient encountered cutaneous and renal adverse events, which resolved upon completion of the trial, indicative of the patient’s receipt of ICIs during the study. Undergoing six cycles of ICIs treatment, the patient underwent monitoring via contrast-enhanced CT or MRI every 3 months post-treatment cessation. Subsequently, the patient experienced intrahepatic recurrence and extrahepatic metastases 1 year after discontinuation of ICIs treatment and 1.5 years post-RFA. Early recurrent HCC nodules frequently exhibit multinodular, diffuse patterns, predominantly involving the liver and often proving nonamenable to surgical interventions (24). Our patient conforms to this profile, presenting with diffuse liver recurrence alongside extensive extrahepatic metastases.

Following a 6-month course of ICIs after RFA, our patient encountered early recurrence 1.5 years after the procedure. The recurrence pattern subsequent to curative HCC treatment is bimodal, with the initial peak typically arising about 1 year post-resection, followed by a second peak at 4–5 years post-resection (2). Despite the preventive administration of ICIs, early recurrence manifested in our patients, prompting further investigation into whether this is attributable to the relatively brief prophylactic period of ICIs application, at least up to 1 year following RFA. The ability of ICIs to engage T lymphocytes endures for a minimum of 20 weeks following cessation of ICIs therapy (25). Furthermore, administering ICIs at low doses subsequent to liver transplantation may serve as a preventive measure against HCC recurrence (26). Additional clinical studies are warranted to discern whether prolonging dosing intervals or reducing ICIs doses could forestall HCC recurrence in high-risk patients.

Several publications have highlighted the treatment of recurrent HCC using ICIs. For some patients with tumors, retreatment with ICIs has proven beneficial. In a phase I/II clinical investigation, 160 patients (including those with various solid tumors) who had discontinued dulvalizumab treatment without disease progression for 1 year were subsequently reintroduced to dulvalizumab upon disease progression. Among the 70 patients who received retreatment, 11.4% exhibited a partial response, 60% maintained stable disease, 22.9% experienced disease progression, and none achieved a complete response (8). Given HCC’s vascular-rich nature, it possesses a unique neovascular architecture that often impedes the accessibility of antitumor medications and immune cells to the tumor site (27). In comparison to late recurrent HCC, early recurrent HCC tends to develop large vessels and possess enhanced microvascular infiltration capability (28). Anti-vascular endothelial growth factor (anti-VEGF) agents have the capacity to normalize tumor blood vessels (29). VEGF triggers the development of myeloid-derived suppressor cells, regulatory T cells, and tumor-associated macrophages, thus contributing to the creation of an immunosuppressive tumor microenvironment (30). Consequently, anti-VEGF agents, by ameliorating the immunosuppressive microenvironment, optimize the efficacy of ICIs treatment, potentially surmounting resistance to ICIs. The IMbrave150 study also demonstrated that the combination of the anti-VEGF agent bevacizumab with the anti-PD-L1 antibody atezolizumab notably enhanced survival among patients with advanced HCC, proving to be significantly superior to sorafenib (7). Hence, despite the patient’s prior history of ICIs treatment, we opted for atezolizumab in conjunction with bevacizumab. Post two cycles of this regimen, the patient’s AFP levels declined rapidly to normal, and a repeated contrast-enhanced CT scan revealed a marked reduction in both intrahepatic and extrahepatic lesions. Following ten cycles of immunotherapy, a contrast-enhanced CT scan indicated an absence of enhancing tumor lesions in the liver, as well as no pulmonary lesions, alongside a significant reduction in adrenal lesions. Subsequent retreatment with ICIs resulted in swift remission for the patient.

## Conclusion

This case indicates that ICIs might postpone the early recurrence of HCC, but an extended duration of ICIs treatment might be necessary to forestall recurrence. Furthermore, for patients with recurrent HCC and a previous history of ICIs therapy, retreatment with ICIs remains a viable option and can still provide benefits.

## Data availability statement

The original contributions presented in the study are included in the article/**Supplementary material**. Further inquiries can be directed to the corresponding author/s.

## Ethics statement

The studies involving humans were approved by The Third Central Hospital of Tianjin Medical Ethics Committee. The studies were conducted in accordance with the local legislation and institutional requirements. The participants provided their written informed consent to participate in this study. Written informed consent was obtained from the individual(s) for the publication of any potentially identifiable images or data included in this article.

## Author contributions

TW: Writing – original draft. FT: Writing – original draft. FL: Data curation, Formal Analysis, Writing – review & editing. WY: Writing – review & editing, Data curation. JL: Writing – review & editing.
