# Supplementary material for: Retreatment with immunotherapy in a patient with hepatocellular carcinoma who received immune checkpoint inhibitors after primary curative treatment: a case report
Source: Front Oncol. 2024 Apr 5;14:1321195. doi: 10.3389/fonc.2024.1321195 (PMC11026608; doi:10.3389/fonc.2024.1321195)
Supplement: Supplementary file 1 [file Table_1.docx]

**Table S1.** Clinical indicators

| Variables | Base line  (May 26, 2020) | Pre-retreatment of ICIs  (June 2, 2022) | The latest tests  (September 15, 2023) |
| --- | --- | --- | --- |
| PT (s) | 13.40 | 13.40 | 12.30 |
| INR | 1.00 | 1.01 | 0.89 |
| WBC (×10⁹/L) | 4.65 | 3.61 | 5.01 |
| NEUT (%) | 63.1 | 72.8 | 63.0 |
| HGB (g/L) | 146 | 108 | 140 |
| PLT (×10⁹/L) | 124 | 113 | 61 |
| ALB (g/L) | 49.3 | 39.1 | 44.9 |
| ALT (U/L) | 23 | 23 | 25 |
| AST (U/L) | 20 | 93 | 26 |
| ALP (U/L) | 67 | 212 | 49 |
| GGT (U/L) | 23 | 201 | 34 |
| TBil (μmol/L) | 22.0 | 23.0 | 21.2 |
| DBIL(μmol/L) | 2.9 | 4.7 | 2.0 |
| LDH (U/L) | 236 | 757 | 221 |
| Cr (mg/dL) | 66 | 68 | 69 |
| AFP(ng/mL) | 170.30 | 1933 | 1.44 |

Abbreviations: ICIs, immune checkpoint inhibitors; PT, prothrombin time; INR, international normalized ratio; WBC, white blood cell; NEUT, neutrophil; HGB, hemoglobin; PLT, platelet; ALB, albumin; ALT, alanine aminotransferase; AST, aspartate aminotransferase; ALP, alkaline phosphatase; GGT, gamma-glutamyltransferase; TBIL, total bilirubin; DBIL, direct bilirubin; LDH, lactate dehydrogenase; Cr, creatinine; AFP, alpha-fetoprotein.
